# Letter to the Editor: “Respiratory complications following minimally invasive esophagectomy: associated factors and predictive value of lung age”

**DOI:** 10.1097/MS9.0000000000005123

**Published:** 2026-05-06

**Authors:** Kanza Zainab, Mamoona Muneer, Muhammad Arham Rafique, Ania Mushtaq

**Affiliations:** a Department of Medicine, Nishtar Medical University, Multan, Pakistan; b Department of Medicine, Gujranwala Medical College, Gujranwala, Pakistan; c Department of Medicine, Mugda Medical College, University of Dhaka, Dhaka, Bangladesh

*Dear Editor,*

We read with considerable interest the manuscript entitled “Respiratory complications following minimally invasive esophagectomy: associated factors and predictive value of lung age” by Pham *et al*^[^1^]^. The study addresses persistent postoperative respiratory complications following minimally invasive esophagectomy (MIE), with particular emphasis on the predictive value of lung age^[^2^]^. However, respiratory complications remain a significant source of postoperative morbidity. A key strength of the study is the incorporation of lung age, calculated using the Japanese Respiratory Society (JRS) formula, as a pragmatic preoperative risk stratification tool^[^3^]^. The findings that older lung age, a greater lung age-real age differential, and recurrent laryngeal nerve palsy were associated with postoperative respiratory complications further underscore the importance of preoperative assessment in the surgical arena.

The authors should be commended for proposing a clinically applicable and noninvasive predictive approach. Esophagectomy is the key stone of esophageal cancer treatment, but morbidity and mortality from anastomotic insufficiency still pose difficulties to patient outcomes^[^4^]^. A recent study demonstrated a remarkably high compliance rate of 82.7% after esophagectomy; this finding highlights the crucial need for multidisciplinary approaches to enhance postoperative outcomes^[^5^]^. Esophagectomy can be helpful in patients with thoracic esophageal squamous cell carcinoma after definitive concurrent chemoradiotherapy, especially in those who have advanced-stage disease. Esophageal cancer has a poor prognosis, and surgery carries the most notable respiratory events, especially pneumonia, being the most common and severe after MIE.

Despite its strengths, several limitations warrant consideration. First, the single-center design and modest sample size (*n* = 70) limit external validity and reduce the robustness of subgroup analyses. Second, the follow-up period of 120 days may underestimate longer-term respiratory sequelae, including chronic aspiration and delayed pulmonary complications. Third, the absence of detailed stratification of pre-existing pulmonary conditions and perioperative variables – such as chronic obstructive pulmonary disease severity, nutritional status, anesthesia protocols, and surgeon experience – introduces potential confounding. Finally, the applicability of the JRS lung age formula to a Vietnamese population remains uncertain, raising concerns regarding measurement validity and generalizability. Collectively, these factors necessitate cautious interpretation when extrapolating the findings to broader clinical settings^[^6^]^.

Secondly, the follow-up duration was limited to 120 postoperative days, and this potentially undervalued long-term respiratory consequences like chronic aspiration, fibrosis, or delayed pulmonary infections^[^7^]^. Given the proven relationship between respiratory complications and long-term survival, it would be worthwhile to investigate these outcomes in subsequent studies.

Thirdly, there was no subclassification of pre-existing pulmonary disease (e.g., chronic obstructive pulmonary disease severity, previous infection, nutrition). These are universally accepted risk factors that may complicate the reported associations. Likewise, perioperative and surgical factors, such as surgeon experience, anesthesia regimen, and compliance with enhanced recovery protocols, were not studied in full, even though they have been shown to substantially impact complication rates ^[^8^]^.

Lastly, the use of the formula for the JRS’s lung age is perhaps not fully tested in the Vietnamese population. This opens up the potential for measurement bias and underscores the importance of population-based validation prior to the clinical implementation of lung age levels in risk prediction.

Finally, although the study offers new evidence that lung age and lung age–real age difference perform better than traditional spirometric measures in the prediction of respiratory complications, these results must be interpreted with caution. Importantly, these limitations collectively constrain the clinical applicability of lung age as a standalone predictive metric and highlight the need for validation within diverse populations and integrated risk models^[^9–12^]^.

In conclusion, Pham *et al* provide valuable insights into the role of lung age in predicting postoperative respiratory complications following MIE^[^13^]^. While the concept is clinically appealing, its current application is limited by methodological constraints and a lack of external validation. Future multicenter studies with longer follow-up and comprehensive risk adjustment are essential to establish lung age as a reliable component of perioperative risk stratification^[^13–15^]^.

TITAN Guidelines: This manuscript is compliant with the TITAN Guidelines, 2025, declaring no use of AI**^[^**16^]^.

## Data Availability

Data available upon request from the authors.
